# Visceral Adipose Tissue Depth as a Novel Predictor for Gestational Diabetes Mellitus: A Comprehensive Meta-Analysis and Systematic Review

**DOI:** 10.3390/medicina60040557

**Published:** 2024-03-29

**Authors:** Weikun Li, Yi Jiang, Ling Feng, Jun Yu

**Affiliations:** Department of Gynecology and Obstetrics, Tongji Hospital, Tongji Medical College, Huazhong University of Science and Technology, Wuhan 430074, China; 13782563048@163.com (W.L.); einsmeer@foxmail.com (Y.J.); fltj007@163.com (L.F.)

**Keywords:** gestational diabetes mellitus, obesity, visceral adipose tissue depth, predictive factor, ultrasound

## Abstract

*Background and Objectives*: The escalating prevalence of gestational diabetes mellitus (GDM) and the limitations associated with utilizing body mass index (BMI) as a predictive measure underscore the imperative need for identifying an optimal early pregnancy predictor. Such a predictor not only mitigates the risk of GDM but also allows for timely implementation of interventions. *Materials and Methods*: This meta-analysis aimed to explore the association between visceral adipose tissue (VAT) depth and the risk of GDM. A thorough search of PubMed, Embase, and Web of Science databases was conducted up to 30 September 2023. The analysis employed a random-effects model to assess the relationship between VAT depth and the likelihood of GDM. *Results*: The inclusion criteria encompassed seven studies involving 1315 women, including 225 diagnosed with GDM. Significantly lower VAT depth was observed in the non-GDM group in comparison to the GDM group (Standardized Mean Difference [SMD]: 0.84; 95% Confidence Interval [CI]: 0.52–1.15; *p* < 0.001). Substantial statistical heterogeneity was noted among studies (I^2^ = 72.88%, *p* = 0.001). Through meticulous sensitivity and subgroup analyses, the source of heterogeneity was identified and thoroughly discussed. Subgroup analyses suggest that different GDM diagnostic criteria and VAT definitions all indicate higher VAT depth in GDM patients during early pregnancy. *Conclusions*: Our findings propose that, during the first trimester, GDM patients exhibit higher VAT depth compared to non-GDM women, highlighting VAT depth as a potential predictive factor for GDM in early pregnancy. This study contributes valuable evidence to the growing body of knowledge surrounding novel predictors for GDM, emphasizing the importance of early intervention strategies.

## 1. Introduction

Gestational diabetes mellitus (GDM) poses a pervasive challenge during pregnancy, characterized by abnormal glucose tolerance first recognized during gestation [1]. This condition carries potential health risks for both mother and fetus [2], and its prevalence has surged by over 30% in numerous countries over the past two decades, imposing substantial global health and economic burdens [3]. Various predisposing factors for GDM have been identified, including advanced maternal age, family history of diabetes, previous GDM, macrosomic baby, non-Caucasian race/ethnicity, being overweight or obesity, and cigarette smoking [4,5]. Notably, being overweight and obesity stand out as significant risk factors, while maternal diet remains one of the few modifiable factors [6].

Despite the prevailing use of body mass index (BMI) as the primary obesity indicator [7], its limitations, such as the inability to distinguish muscle mass from bone and fat mass, have prompted criticism [8]. It has been documented that the preferential storage of fat in central rather than lower-body depots in obese pregnancy leads to lipotoxicity. The combination of excess fatty acids and oxidative stress leads to the production of oxidized lipids, which can be cytotoxic and influence gene expression by acting as ligands for nuclear receptors [9]. Hence, maternal central obesity, as indicated by visceral adipose tissue (VAT) depth, emerges as a potentially superior marker compared to BMI for predicting the onset of obesity-related diseases. VAT depth, evaluated as a measure of central obesity, offers heightened accuracy and precision over waist circumference (WC) or waist–hip ratio (WHR), proving to be a convenient, fast, and routinely utilized method during pregnancy.

Studies, such as those by Alves et al. [10] and Thaware et al. [11], have underscored the predictive superiority of VAT depth over prepregnancy BMI in anticipating GDM. Furthermore, the utilization of ultrasound to measure abdominal VAT depth in early pregnancy has been associated with enhanced sensitivity in selectively screening for GDM [10,12].

While the link between central obesity and GDM has garnered attention in previous research, the specific correlation between VAT depth and GDM remains inadequately explored [10,11,12,13,14,15,16]. This study proposes a hypothesis: early pregnancy ultrasound detection of VAT can serve as a predictive indicator for GDM. To validate this hypothesis, we systematically included cohort studies and conducted a meta-analysis to comprehensively investigate the relationship between VAT depth and the risk of GDM.

This study is not the first to propose the predictive value of VAT in GDM. A review published last year provided a detailed overview of the role of early pregnancy fat tissue thickness in predicting GDM [17]. However, due to variations in VAT definitions and GDM diagnostic criteria among different studies, and the relatively small sample sizes in most studies, there is considerable heterogeneity. Previous meta-analyses primarily focused on the relationship between central obesity and GDM, which is influenced by subcutaneous fat thickness and involves multiple indicators, making clinical detection challenging. In contrast, this study focuses specifically on the independent predictive value of VAT. By comparing the difference in VAT between GDM patients and normal pregnant women in early pregnancy, we can further demonstrate the predictive value of VAT for GDM. Our findings contribute to advancing our understanding of GDM predictors and highlight the potential of VAT depth as a valuable and precise predictor in the realm of gestational diabetes research.

## 2. Method

### 2.1. Data Sources and Search Strategy

Adhering to the guidelines outlined by the Preferred Reporting Items for Systematic Reviews and Meta-Analyses (PRISMA) statement [18] (Supplementary Material S1), we conducted a meticulous meta-analysis. Our study also followed the Cochrane Handbook for Systematic Reviews of Interventions and was registered in the International Prospective Register of Systematic Reviews (registration number: CRD42024507569). Our search spanned PubMed, Embase, and Web of Science, encompassing medical literature published from inception to 30 September 2023, irrespective of country, but excluding books, guidelines, and case reports. We employed a comprehensive set of keywords and MeSH terms, including gestational diabetes, abdominal obesity, subcutaneous fats, body fat distribution, and visceral adipose tissue. The specific search strategy can be found in Supplementary Material Table S1. Additionally, we scrutinized prior meta-analyses and related reviews pertinent to our research question, ensuring the identification of studies not captured by our initial search strategy.

### 2.2. Inclusion Criteria and Exclusion Criteria

Inclusion criteria for the meta-analysis were defined using the PICOS (Population, Intervention, Comparison, Outcome, and Study design) evidence-based question framework as follows: (1) population: pregnant female volunteers; (2) intervention: ultrasound examinations with measurement of VAT depth; (3) comparison: not applicable (since this is a meta-analysis, there may not be a specific comparison group); (4) outcome: diagnosis of GDM based on oral glucose tolerance test (OGTT) results; (5) study design: cohort studies. Additionally, eligible studies were required to provide detailed data on VAT depth for each study group.

Exclusion criteria for the meta-analysis were applied to studies exhibiting the following characteristics: (1) being non-English-language medical literature; (2) inclusion of study subjects previously diagnosed with diabetes (type 1 or 2) or with a history of gestational diabetes; (3) absence of reported mean, standard deviation (SD), or confidence interval (CI) values for VAT depth; and (4) identification as case reports or previously published systematic reviews and meta-analyses.

### 2.3. Data Extraction and Quality Assessment

Duplicate records were removed during the initial screening using EndNote reference management software. Two reviewers (WKL and YJ) participated in the initial screening process, which involved screening the titles and abstracts of each study for eligibility. Subsequently, full-text articles were obtained for all studies deemed to meet the inclusion criteria. Another set of two reviewers (JY and LF) screened the full-text articles, and any study found to meet the exclusion criteria based on the full text was excluded. In cases where there was a disagreement between the two reviewers in a group, the final decision on whether to include the record was made by the other set of two reviewers. The extracted information from each study included the author’s name, year of publication, study design, study population characteristics, number of subjects, examination period, GDM diagnosis criteria, definition of VAT depth, and the corresponding mean, standard deviation (SD), or confidence interval (CI) values for VAT depth.

To assess the quality of the studies, the Newcastle–Ottawa quality assessment scale (NOS) for cohort studies was employed. In adherence to NOS guidelines, modified studies that attained five or more stars were classified as high-quality for inclusion in the meta-analysis. This rigorous quality assessment ensures the reliability and robustness of the synthesized evidence in our meta-analytical findings.

### 2.4. Data Synthesis and Statistical Analysis

The data synthesis and statistical analyses were conducted using STATA 16.0. Random-effects models were utilized for analyses exhibiting high heterogeneity, whereas fixed-effects models were applied for analyses showing low heterogeneity to derive summary estimates, elucidating the relationship between VAT depth and the risk of GDM. The heterogeneity between studies was assessed using P heterogeneity, Cochran’s Q test, and Higgins I^2^. Significant heterogeneity was considered present if P_heterogeneity_ < 0.05, I^2^ > 50%, and P_Cochran’s Q test_ > 0.1. The results were expressed as standard mean differences (SMDs) with corresponding 95% CIs.

To identify the source of heterogeneity, RevMan 5.4 was utilized by systematically excluding each study one at a time. Subgroup analyses were performed, taking into account various factors, including study design, number of participants, number of cases, trimester, GDM diagnostic criteria, and the definition of VAT depth. These meticulous approaches were employed to comprehensively explore and understand potential sources of variation across the included studies, enhancing the robustness and interpretability of our meta-analytical results.

## 3. Results

### 3.1. Study Selection and Evaluation

Initially, we identified a total of 1024 potential articles from the three databases. Following the removal of duplicates and the application of exclusion criteria based on titles and abstracts, 34 studies underwent a comprehensive review through full-article assessment for eligibility. Ultimately, seven articles were deemed eligible for inclusion in our meta-analysis. The exclusion criteria encompassed studies that solely measured maternal subcutaneous ultrasound thickness (7 studies), those lacking essential data such as (SD or 95% CI (10 studies), non-English language publications (1 study), assessments focused on fetal outcomes (7 studies), and editorial articles (2 studies).

**Figure 1:**
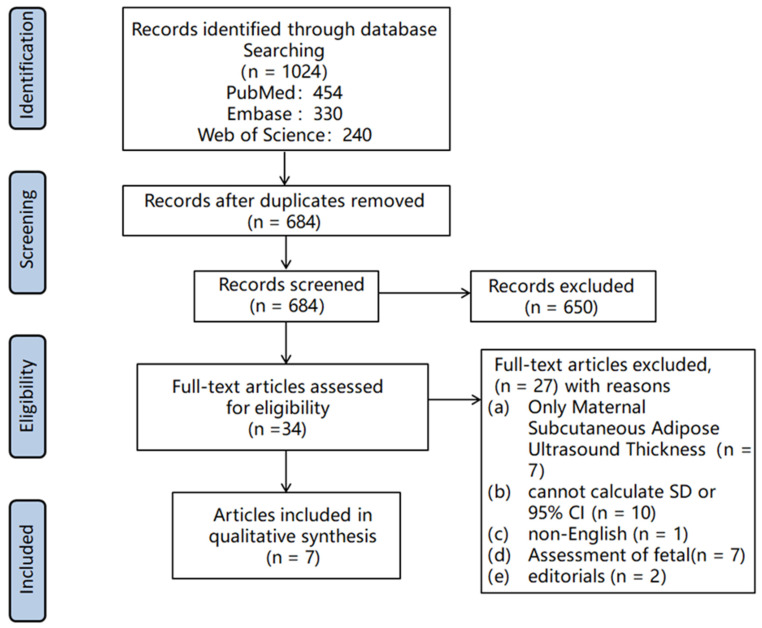
Flow chart of study selection.

The final analysis incorporated a total of 1315 participants, among whom 225 were diagnosed with GDM (Figure 1). This rigorous selection process ensures the reliability and relevance of the studies included in our meta-analysis, enhancing the robustness of our findings.

### 3.2. Characteristics and Quality Assessment of Included Studies

The essential characteristics of each study are summarized in Table 1. Among the seven studies included, there were 225 pregnant women diagnosed with GDM and 1090 non-GDM pregnant women. Six of these studies [10,11,12,14,15,16] were prospective cohort studies, while one [13] was a case-control study. The ultrasound examinations in six studies [10,11,12,14,15,16] were conducted at ≤20 weeks, whereas one study [13] performed the ultrasound at the gestational age of 24–28 weeks. All studies employed the oral glucose tolerance test (OGTT) for GDM diagnosis, with only one study [12] utilizing its own diagnostic criteria, while the others [10,11,13,14,15,16] adhered to the International Association of Diabetes and Pregnancy Study Groups (IADSPG) screening criteria.

The definition of VAT depth varied among studies: three studies [12,13,16] defined VAT depth as the distance between the liver surface and the linea alba, while four studies [10,11,14,15] defined VAT depth as the distance between the linea alba and the anterior aspect of the abdominal aorta.

The Newcastle–Ottawa Scale (NOS) was employed to assess the quality of the included studies, and all studies achieved five or more stars, indicating a high level of quality (Table 2). This quality assessment reinforces the credibility and reliability of the studies included in our meta-analysis.

### 3.3. The Relation between VAT Depth and the Risk of GDM

**Figure 2:**
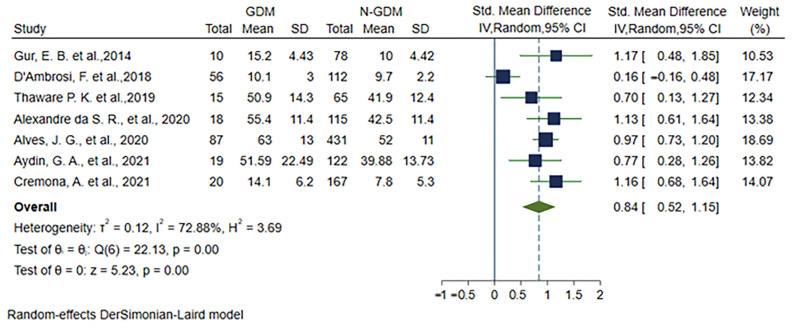
Forest plots of studies on the relationship between VAT depth and the risk of GDM [10,11,12,13,14,15,16].

We have synthesized and visualized the relationship between VAT depth and the risk of GDM in pregnant women, as depicted in the forest plot (Figure 2). The analysis reveals a significant difference in VAT depth between the non-GDM and GDM groups, with a SMD of 0.84 (95% CI: 0.52–1.15; *p* < 0.001). However, it is noteworthy that a considerable degree of statistical heterogeneity exists among the studies (I^2^ = 72.88%, *p* = 0.001).

Given the observed heterogeneity, we proceeded with sensitivity analysis and subgroup analysis to pinpoint the potential sources of variation in our findings. This rigorous examination aims to enhance the robustness and reliability of our meta-analysis by identifying and addressing factors contributing to the observed heterogeneity.

### 3.4. Sensitivity Analysis and Subgroup Analysis

**Figure 3:**
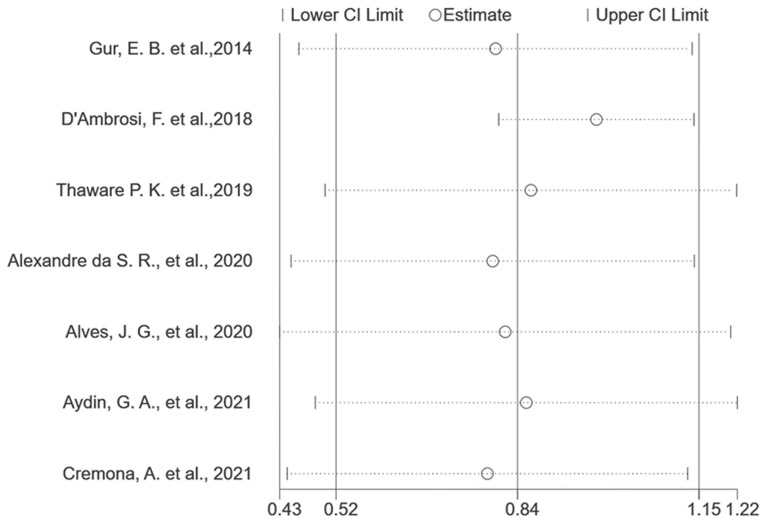
Sensitivity analysis of included studies [10,11,12,13,14,15,16].

We conducted a sensitivity analysis by systematically removing one included study at a time to investigate the influence of each study on the overall merged effect, as illustrated in Figure 3 and summarized in Table 3. While Figure 3 suggests that the meta-analysis results remained stable and reliable, a noteworthy observation is that the I^2^ decreased to 0 (*p* = 0.73) when one study [13] was excluded, as indicated in Table 3.

The summary of subgroup analyses is presented in Table 4. Stratifying by factors such as the number of participants, number of cases, GDM diagnostic criteria, or the definition of VAT depth did not reveal significant heterogeneity among respective subgroups (*p* ** > 0.05), indicating that these factors are not contributing to the overall heterogeneity. However, significant heterogeneity was observed in some subgroups (number of participants > 100: I^2^ = 80.8%, *p* = 0.000; number of cases > 50: I^2^ = 93.6%, *p* = 0.000; using IADSPG screening criteria: I^2^ = 76.1%, *p* = 0.001; and VAT defined as the distance between the liver surface and the linea alba: I^2^ = 86.7, *p* = 0.001). Importantly, all these subgroups include the same study [13]. Furthermore, when stratifying by study design and examination period, significant heterogeneity among the subgroups was observed (*p* ** = 0.000). Since the subgroup “case-control study” and the subgroup “gestational age > 20 weeks” share only one common study [13], aligning with the findings from the sensitivity analysis, we posit that study [13] is a potential source of the observed heterogeneity.

### 3.5. Results after Omitting the Heterogeneous Article

**Figure 4:**
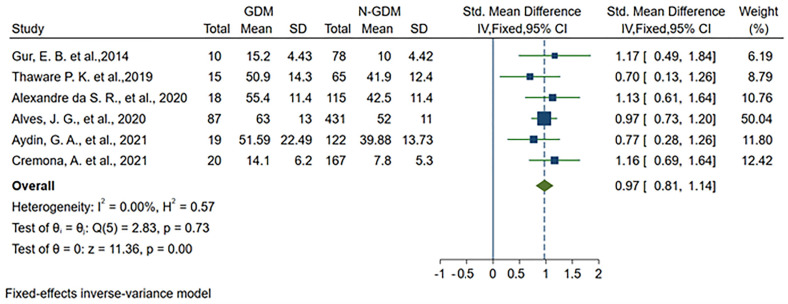
Forest plots of studies on the relationship between VAT depth and the risk of GDM after omitting the heterogeneous study [10,11,12,14,15,16].

Having identified the article responsible for the observed heterogeneity, we proceeded with the analysis anew. The forest plot is depicted in Figure 4, and the summary of subgroup analyses is presented in Table 5. Given that all included studies were cohort studies and the I^2^ was now below 50%, we employed a fixed-effects inverse-variance model for this iteration. Consistent with the prior results, the VAT depth of the N-GDM group remained significantly lower compared to the GDM group (SMD: 0.97; 95% CI: 0.81–1.14; *p* < 0.001).

The subgroup analyses revealed no significant heterogeneity within each subgroup, reaffirming the stability and reliability of the findings after addressing the potential source of heterogeneity. This refined analysis enhances the robustness of our results and provides greater confidence in the validity of the observed relationship between VAT depth and the risk of GDM in pregnant women.

## 4. Discussion

Our meta-analysis, involving 1315 pregnant women, establishes a direct correlation between VAT depth and the risk of developing GDM. Notably, healthy women not diagnosed with GDM exhibit a lower VAT depth compared to those diagnosed with GDM.

Regarding the interpretability of the SMDs in our results, we acknowledge the challenge in assessing the clinical significance of these differences. While the SMDs provide a measure of effect size, translating these values into tangible clinical implications can be complex. Despite this challenge, our study consistently demonstrates a significant role for VAT depth in predicting the risk of GDM. The observed differences in VAT depth between women diagnosed with GDM and those without provide robust evidence of the association between visceral adiposity and GDM risk. This suggests that while the precise interpretation of SMDs may pose difficulties, the overarching message regarding the importance of VAT depth in GDM prediction remains clear and impactful. Further exploration into the clinical relevance of these findings, perhaps through additional analyses or prospective studies, may help elucidate the practical implications of the observed SMDs.

Specifically, one study [19] emphasized visceral adiposity as a more potent risk factor for GDM compared to other obesity phenotypes, highlighting the critical link between visceral adiposity and GDM. Previous research [20,21] has demonstrated a robust relationship between visceral adiposity and insulin resistance patterns in glucose homeostasis, extending to type 2 diabetes mellitus [22]. The enhanced catecholamine-induced lipolysis in visceral tissue, leading to increased fatty acid delivery to the hepatic portal circulation, may adversely impact in vivo insulin action [23,24]. Additionally, VAT, recognized as a key indicator of visceral adiposity, is easily and accurately measured with ultrasound [25]. A study [26] using VAT measured with ultrasound reported the highest odds ratio (OR) for central obesity (OR: 4.69, 95% CI: 0.99–22.16), consistent with our findings.

Despite the meaningful result, significant heterogeneity was observed. After thorough sensitivity and subgroup analyses, an article, [13], was identified as the source of heterogeneity. This prospective case-control study measured adipose thickness with transabdominal ultrasound at 24–28 weeks’ gestation, suggesting that ultrasound timing plays a key role in this difference. Notably, a study, [27], indicated a tendency towards decreased subcutaneous adipose tissue and increased preperitoneal adipose tissue accumulation during pregnancy, providing evidence that VAT measurement in the second trimester may be less effective in predicting GDM due to VAT growth. Consequently, we assert that VAT measurement in early pregnancy is essential for assessing GDM risk, with better predictive efficacy than measurement in the second trimester. Study design may also contribute to heterogeneity due to potential biases.

As mentioned earlier, BMI may have limitations in predicting GDM due to its inability to distinguish between muscle mass, bone mass, and fat mass. In fact, in three out of the seven studies included in this meta-analysis, the results directly indicated that VAT has a higher predictive value for GDM than BMI [10,12,14]. Other potential predictors include indicators reflecting central obesity, such as waist circumference and waist-to-hip ratio. Central obesity is also considered a good indicator for predicting GDM, and both VAT and waist circumference or waist-to-hip ratio can, to some extent, reflect central obesity [26]. However, waist circumference and waist-to-hip ratio are also influenced by subcutaneous fat thickness [12]. Nevertheless, a study involving 1106 patients suggested that subcutaneous fat thickness cannot effectively predict diabetes in women, but reducing visceral fat can reduce the risk of type 2 diabetes in women [28]. Another study showed that abdominal fat thickness is an independent risk factor for type 2 diabetes [29], possibly due to the increase in insulin resistance in muscle tissue caused by free fatty acids and cytokines produced by abdominal fat [30,31]. Although these studies focused on populations with type 2 diabetes, given the similarity between GDM and type 2 diabetes, and the fact that women with a history of GDM have a significantly increased risk of developing type 2 diabetes even after delivery [32,33], we still believe that VAT is the most important parameter for predicting GDM. However, using multiple indicators such as VAT combined with waist circumference, waist-to-hip ratio, BMI, etc., to construct a predictive model is feasible.

Subsequent analyses after omitting the heterogeneous article demonstrated a consistently lower VAT depth in the non-GDM group compared to the GDM group. In subgroup analyses, differences in GDM diagnostic criteria and VAT definitions were not pronounced. However, after excluding the heterogeneous article, a subtle difference emerged between VAT definitions, suggesting that the former definition (distance between the liver surface and the linea alba) may have a slightly better predictive effect. Further research is needed to confirm this potential distinction.

This study demonstrates the feasibility of using VAT to predict GDM risk in early pregnancy, offering implications for medical management to effectively reduce GDM occurrence. Early prediction becomes crucial, as GDM diagnosis typically relies on oral glucose tolerance tests at 24–28 weeks, leaving limited time for lifestyle interventions to take effect. In addition, there is currently a lack of a unified definition for VAT. More precisely, VAT refers to visceral adipose tissue, which is located within the abdominal cavity surrounding multiple organs, and ultrasonographers may vary in their measurement practices. However, due to the lack of standardized measurement criteria, studies in this area lack consistency and are challenging to translate into practical applications. Based on our subgroup analysis results, we recommend the use of the distance between the liver surface and the linea alba as the definition for VAT depth, as it has demonstrated higher accuracy in predicting GDM. We also suggest measuring VAT depth during nuchal translucency (NT) examination in early pregnancy, as NT examination is the first prenatal screening test and is performed relatively early, approximately ten weeks before the OGTT test. This allows for early identification of high-risk GDM patients for lifestyle interventions, making it an appropriate time point for screening.

The strengths of this meta-analysis lie in its systematic exploration, providing valuable insights for clinical decision making, and the inclusion of high-quality articles with Newcastle–Ottawa Scale scores exceeding 5 stars. However, this study has several limitations. Firstly, the VAT outcome exhibited high heterogeneity, likely attributed to variations in study durations, patient characteristics, and control groups. Secondly, publication bias was not evaluated due to the small number of included studies, potentially impacting the robustness of our findings. Data insufficiency led to the exclusion of some high-quality articles, and the omission of data from the literature reporting abnormal glucose tolerance without meeting GDM diagnostic criteria resulted in limited data collection. Although a correlation between VAT depth and GDM risk was demonstrated, the specific VAT depth threshold for preventive interventions remains undetermined, warranting attention in future studies.

## 5. Conclusions

In conclusion, our meta-analysis reveals that GDM patients exhibit higher VAT depth in the first trimester compared to non-GDM women. This significant finding holds particular relevance considering the escalating prevalence of GDM. The observed association between GDM and increased VAT depth during early pregnancy provides a practical and valuable clue that could contribute to the development of preventive strategies for GDM.

## Figures and Tables

**Table 1:** Characteristics of studies included in the meta-analysis.

Author	Study Design	Country	Study Size	Gestational Week	GDM Diagnosis	Definition of VAT	Visceral Adipose Tissue Depth ± SD (mm)
Gur et al., 2014 [12]	Prospective cohort study	Turkey	10/88	4–14 weeks	Other	Distance between the liver surface and the linea alba	GDM: 15.2 ± 4.43
							N-GDM: 10 ± 4.42
D’Ambrosi et al., 2018 [13]	case-control study	Italy	56/168	24–28 weeks	IADSPG	Distance between the liver surface and the linea alba	GDM: 10.1 ± 3
							N-GDM: 9.7 ± 2.2
Thaware et al., 2019 [11]	Prospective cohort study	UK	15/80	9–18 weeks	IADSPG	Distance between the linea alba and the anterior aspect of the abdominal aorta	GDM: 50.9 ± 14.3
							N-GDM: 41.9 ± 12.4
Rocha et al., 2020 [14]	Prospective cohort study	Brazil	18/133	≤20 weeks	IADSPG	Distance between the linea alba and the anterior aspect of the abdominal aorta	GDM: 55.4 ± 11.4
							N-GDM: 42.5 ± 11.4
Alves et al., 2020 [10]	Prospective cohort study	Brazil	87/518	at a mean of 14.4 weeks	IADSPG	Distance between the linea alba and the anterior aspect of the abdominal aorta	GDM: 63 ± 13
							N-GDM: 52 ± 11
Aydin et al., 2021 [15]	Prospective cohort study	Turkey	19/141	11–14 weeks	IADSPG	Distance between the linea alba and the anterior aspect of the abdominal aorta	GDM: 51.59 ± 22.49
							N-GDM: 39.88 ± 13.73
Cremona et al., 2021 [16]	Prospective cohort study	Ireland	20/187	10–16 weeks	IADSPG	Distance between the liver surface and the line alba	GDM: 14.1 ± 6.2
							N-GDM: 7.8 ± 5.3

IADSPG: International Association of Diabetes and Pregnancy Study Groups; VAT: Visceral adipose tissue; GDM: Gestational diabetes mellitus; N-GDM: Non-Gestational diabetes mellitus.

**Table 2:** Quality assessment of the cohort and cross-sectional studies included in the meta-analysis using the Newcastle–Ottawa Scale (NOS).

Study ID	Selection				Comparability of Cohorts on the Basis of the Design or Analysis	Outcome		
	Representativeness of the Exposed Cohort	Selection of the Nonexposed Cohort	Ascertainment of Exposure	Demonstration That Outcome of Interest Was Not Present at Start of Study		Assessment of Outcome	Was Follow-up Long Enough for Outcomes to Occur	Adequacy of Follow-up of Cohorts
Gur et al., 2014 [12]	☆	☆	☆	☆	☆☆	☆	☆	☆
D’Ambrosi et al., 2018 [13]	☆	☆	☆	-	☆	☆	-	☆
Thaware et al., 2019 [11]	☆	☆	☆	☆	☆☆	☆	☆	☆
Alexandre et al., 2020 [14]	☆	☆	☆	☆	☆☆	☆	☆	☆
Alves et al., 2020 [10]	☆	☆	☆	☆	☆☆	☆	☆	☆
Aydin et al., 2021 [15]	☆	☆	☆	☆	☆	☆	☆	☆
Cremona et al., 2021 [16]	☆	☆	☆	☆	☆☆	☆	☆	☆

☆: Each “☆” symbol represents one point in the Newcastle–Ottawa Scale (NOS).

**Table 3:** The influence of each study on I^2^.

Eliminate the Study	The Variation of I^2^ (%)	*p*
Gur et al., 2014 [12]	76	0.0008
D’Ambrosi et al., 2018 [13]	0	0.73
Thaware et al., 2019 [11]	77	0.0005
Alexandre et al., 2020 [14]	76	0.001
Alves et al., 2020 [10]	74	0.002
Aydin et al., 2021 [15]	77	0.0005
Cremona et al., 2021 [16]	75	0.001
Total	73	0.001

**Table 4:** Summary result of the subgroup analyses.

	VAT Depth					
	No. of Studies	SMD	95% CI	I^2^ (%)	*p* *	*p* **
Overall studies	7	0.84	0.52–1.15	72.88	0.001	
Subgroup analyses						
Study Design						0.000
Prospective cohort study	6	0.97	0.81–1.14	0	0.732	
Case-control study	1	0.16	−0.16–0.48	NA	NA	
No. of participants						0.804
≤100	2	0.89	0.44–1.35	5.7	0.303	
>100	5	0.82	0.43–1.21	80.8	0.000	
No. of cases						0.331
≤50	5	0.98	0.74–1.22	0	0.594	
>50	2	0.57	−0.22–1.36	93.6	0.000	
Period						0.000
≤20 weeks	6	0.97	0.81–1.14	0	0.732	
>20 weeks	1	0.16	−0.16–0.48	NA	NA	
GDM diagnosis						0.346
Other	1	1.17	0.48–1.85	NA	NA	
IADSPG	6	0.80	0.46–1.14	76.1	0.001	
Definition of VAT						0.737
Distance between the liver surface and the linea alba	3	0.80	0.04–1.55	86.7	0.001	
Distance between the linea alba and the anterior aspect of the abdominal aorta	4	0.93	0.74–1.12	0	0.64	

VAT: visceral adipose tissue depth; GDM: gestational diabetes mellitus; SMD: standard mean difference; CI: confidence interval; NA: not available; *p* *: *p*-value for heterogeneity within each subgroup; *p* **: *p*-value for heterogeneity between subgroups.

**Table 5:** Summary result of the subgroup analyses after omitting the heterogeneous article.

	VAT Depth					
	No. of Studies	SMD	95% CI	I^2^ (%)	*p* *	*p* **
Overall studies	6	0.97	0.81–1.14	0	0.732	
Subgroup analyses						
No. of participants						0.691
≤100	2	0.89	0.45–1.33	5.7	0.303	
>100	4	0.99	0.81–1.17	0	0.665	
No. of cases						0.938
≤50	5	0.98	0.74–1.22	0	0.594	
>50	1	0.97	0.74–1.22	NA	NA	
GDM diagnosis						0.568
Other	1	1.17	0.48–1.85	NA	NA	
IADSPG	5	0.96	0.79–1.14	0	0.651	
Definition of VAT						0.293
Distance between the liver surface and the linea alba	2	1.16	0.77–1.56	0	0.993	
Distance between the linea alba and the anterior aspect of the abdominal aorta	4	0.93	0.74–1.12	0	0.640	

VAT: visceral adipose tissue depth; GDM: gestational diabetes mellitus; SMD: standard mean difference; CI: confidence interval; NA: not available; *p* *: *p*-value for heterogeneity within each subgroup; *p* **: *p*-value for heterogeneity between subgroups.

## Data Availability

All data generated or analyzed during this study are included in this article. Further enquiries can be directed to the corresponding author.

## Supplementary Materials

The following supporting information can be downloaded at: https://www.mdpi.com/article/10.3390/medicina60040557/s1. Supplementary Material S1: PRISMA 2020 Checklist, Reference [34] is cited in the supplementary materials. Table S1: The search strategy in PubMed, Embase and Web of Science of the relationship between “Central obesity” and “Gestational diabetes mellitus”.
