# Supplementary material for: Visceral Adipose Tissue Depth as a Novel Predictor for Gestational Diabetes Mellitus: A Comprehensive Meta-Analysis and Systematic Review
Source: Medicina (Kaunas). 2024 Mar 29;60(4):557. doi: 10.3390/medicina60040557 (PMC11052462; doi:10.3390/medicina60040557)
Supplement: Supplementary file 1 [file medicina-60-00557-s001.zip › Supplementary material Table S1.pdf]

Supplement Table S1. The search strategy in PubMed, Embase and Web of Science of the relationship between "Central obesity" and "Gestational diabetes mellitus"

#1	Search "Obesity, Abdominal"[MeSH]/[exp]
#2	"Obesity, Abdominal" OR "Abdominal Obesities" OR "Obesities, Abdominal" OR "Abdominal Obesity" OR "Central Obesity" OR "Central Obesities" OR "Obesities, Central" OR "Obesity, Central" OR "Obesity, Visceral" OR "Visceral Obesity" OR "Obesities, Visceral" OR "Visceral Obesities"
#3	Search "Waist Circumference"[MeSH]/ [exp]
#4	"waist circuit" OR "waistline"
#5	Search "Waist-Hip Ratio"[MeSH]
#6	"Ratio, Waist-Hip" OR "Ratios, Waist-Hip" OR "Waist Hip Ratio" OR "Waist-Hip Ratios" OR "Waist-to-Hip Ratio" OR "Ratio, Waist-to-Hip" OR "Ratios, Waist-to-Hip" OR "Waist to Hip Ratio" OR "Waist-to-Hip Ratios"
#7	Search "Body Fat Distribution"[MeSH]/[exp]
#8	"Distribution, Body Fat" OR "Fat Distribution, Body" OR "Body Fat Patterning" OR "Fat Patterning, Body" OR "Patterning, Body Fat" OR "Body Fat Index"
#9	#2 OR #4 OR #6 OR #8
#10	Search "Pregnancy"[MeSH]/[exp]
#11	"gestation" OR "gestational" OR "pregnant women" OR "gravidity" OR "pregnancies"
#12	Search "Diabetes, Gestation"[MeSH]/[exp]
#13	"blood glucose" OR "glucose metabolism" OR "glucose tolerance" OR "glucose intolerance" OR "hyperglycemia" OR "gestational diabetes" OR "gestational diabetes mellitus" OR "pregnancy-induced diabetes" OR "diabetes, pregnancy-induced" OR "diabetes, pregnancy induced" OR "diabetes mellitus, gestational" OR "pregnancy in diabetes" OR "Diabetes"
#14	#9 AND #11 AND #13
